# Biomarkers of Alzheimer's disease pathophysiology and delirium

**DOI:** 10.1016/j.ebiom.2026.106252

**Published:** 2026-04-10

**Authors:** Kristi Henjum, Bjørn Erik Neerland, Kaj Blennow, Henrik Zetterberg, Christian Thomas Pollmann, Roy Bjørkholt Olsen, Lene Solberg, Marius Myrstad, Olav Tobias Ødegaard, Adi Karabeg, Alan Gulestøl, Ane Victoria Idland, Leiv Otto Watne

**Affiliations:** a Oslo Delirium Research Group, Institute of Clinical Medicine, University of Oslo, 0318, Oslo, Norway; b Department of Geriatric Medicine, Oslo University Hospital, 0424, Oslo, Norway; c Department of Psychiatry and Neurochemistry, Institute of Neuroscience and Physiology, The Sahlgrenska Academy, University of Gothenburg, Gothenburg, Sweden; d Clinical Neurochemistry Laboratory, Sahlgrenska University Hospital, Mölndal, Sweden; e Paris Brain Institute, ICM, Pitié-Salpêtrière Hospital, Sorbonne University, Paris, France; f Neurodegenerative Disorder Research Center, Division of Life Sciences and Medicine, Department of Neurology, Institute On Aging and Brain Disorders, University of Science and Technology of China and First Affiliated Hospital of USTC, Hefei, PR China; g Department of Neurodegenerative Disease, UCL Institute of Neurology, London, UK; h UK Dementia Research Institute at UCL, London, UK; i Hong Kong Center for Neurodegenerative Diseases, Hong Kong Special Administrative Region of China; j Wisconsin Alzheimer's Disease Research Center, University of Wisconsin School of Medicine and Public Health, University of Wisconsin–Madison, Madison, WI, USA; k Department of Orthopedic Surgery, Akershus University Hospital, Oslo, Norway; l Department of Anesthesiology and Intensive Care, Sørlandet Hospital, Arendal, Norway; m Division of Orthopaedic Surgery, Oslo University Hospital, 0424, Oslo, Norway; n Department of Internal Medicine, Bærum Hospital, Vestre Viken Hospital Trust, Bærum, Norway; o Department of Anesthesiology, Akershus University Hospital, Kongsvinger, Norway; p Department of Orthopedic Surgery, Akershus University Hospital, Kongsvinger, Norway; q Department of Anaesthesiology, Oslo University Hospital, Oslo, Norway; r Department of Anaesthesiology, Akershus University Hospital, Norway; s Department of Geriatric Medicine, Akershus University Hospital, 1478, Lørenskog, Norway

**Keywords:** Delirium, Alzheimer's disease, CSF biomarkers, Acute hip fracture, Dementia

## Abstract

**Background:**

Alzheimer's disease (AD) is characterised by amyloid-beta (A) and tau (T) pathology, and neurodegeneration (N). AT(N) neuropathology precedes the symptomatic presentation of the disease. This asymptomatic phase may present vulnerability towards delirium, but studies are inconclusive. In this cross-sectional study of hip fracture patients, we aimed to explore the association between AD biomarkers for AT(N) neuropathology and delirium, with focus on patients without clinical dementia.

**Methods:**

The AT(N) biomarkers were analysed in cerebrospinal fluid (CSF) in hip fracture patients (n = 401). Pre-fracture dementia was defined by IQCODE ≥3.44 (n = 164). Delirium was diagnosed according to the DSM-5 criteria. The CSF concentrations of amyloid-beta_1-42_ (Aβ42), amyloid-beta_1-40_ (Aβ40), phosphorylated tau_181_ (p-tau) and total-tau (t-tau) were quantified by Lumipulse G assays (Fujirebo, Ghent, Belgium). The Aβ42/Aβ40 ratio (cutoff A+ <0.72), p-tau_181_ (T+ >50 pg/ml) and t-tau (N+ >409 pg/ml) were used to determine A, T and N status, respectively.

**Findings:**

Hip fracture patients with delirium had lower CSF Aβ42 concentrations and higher concentrations of CSF p-tau and CSF t-tau than those without delirium (student's t-test, all p-values <0.001). Brain Aβ-deposition, A+ was more common in patients with delirium (χ^2^ p < 0.001). In patients without pre-fracture dementia, delirium was associated with lower CSF Aβ42 concentrations (student's t-test, p = 0.005), and higher CSF concentrations of p-tau (student's t-test p = 0.004) and t-tau (student's t-test p = 0.002). A higher proportion developed delirium among those A+T+ (33%) compared to A−T− (17%, χ^2^ p = 0.02).

**Interpretation:**

These findings support that the AD AT neuropathology is a risk factor for delirium in patients without clinical dementia.

**Funding:**

South-Eastern Norway Regional Health Authorities (# 2017095), the Norwegian Health Association (#16149, #19536, #1513) and by Wellcome Leap's Dynamic Resilience Program (jointly funded by Temasek Trust) (#104617).

**Research in context**

**Evidence before this study**

PubMed and Web of Science were searched using the terms “delirium”, “cerebrospinal fluid”, “biomarkers” “Alzheimer's disease”, “hip fracture” in varying combinations until January 2026. Original research articles were further assessed. The search was not restricted by language, but primarily articles in English were included due language barriers.

**Added value of this study**

Delirium describes a temporary acute neuropsychiatric syndrome precipitated by acute somatic illness or a trauma. People with dementia are vulnerable towards delirium and a delirium is risk factor for subsequent cognitive decline. Alzheimer's disease (AD) is the most common cause of dementia. Increased knowledge of the delirium pathophysiology is of value as delirium can be traumatic to the patient and is associated with prolonged hospital stays, subsequent incident dementia and progression of an existing dementia. Studies examining Alzheimer's disease AT(N) neuropathophysiology have either been of limited size or have been performed prior to the common introduction of the AT(N) system and the results are inconsistent.

**Implications of all the available evidence**

Our findings support that the AD AT neuropathology is a risk factor for delirium in patients without clinical dementia. This study in patients with hip fracture corroborates in particular an association between brain amyloid-beta deposition, an early event in Alzheimer's disease neuropathophysiology, and delirium.

## Introduction

Delirium describes a temporary acute neuropsychiatric syndrome precipitated by acute somatic illness or a trauma.^1^ People with dementia are more vulnerable towards delirium,^2^^,^^3^ regardless of the precipitating factor. Dementia and delirium are also connected as delirium is a risk factor for subsequent cognitive decline, including dementia.^4^^,^^5^ Better understanding of the delirium pathophysiology and the delirium risk factors could benefit the development of pharmacological prevention strategies. Delirium prevention could potentially be of huge importance since delirium is associated with prolonged hospital stays and subsequent incident dementia and progression of an existing dementia.^6^^,^^7^

Alzheimer's disease (AD) is the most common cause of dementia.^8^ Extracellular amyloid plaques (A), intracellular neurofibrillary tangles (NFTs) of tau (T) protein and neurodegeneration (N) with brain atrophy in certain brain regions defines the AD neuropathology.^9^ The neuropathology evolves over decades, preceding the clinical disease in a preclinical phase.^10^^,^^11^ The AD amyloid-beta and tau pathologies are accompanied by neuroinflammation^11^ which is speculated to increase the vulnerability towards delirium.^12^ The AT(N) status may be determined *in vivo* with high accuracy using biomarkers.^13^^,^^14^ Thereby cognitively unimpaired people with brain AD–type pathology also termed preclinical AD, may be identified and patients classified according to their underlying AT(N) pathology.^13^ Studies point to an association between the AD AT(N) neuropathology and delirium, with an increased risk for delirium also in patients without clinical dementia.^15^, ^16^, ^17^, ^18^ However, most patients in these studies were not classified accordring to the AT(N) classification since the studies were performed prior or around the time this classification was brought into common use. Relatively few patients in these studies developed delirium since only Idland et al.^15^ included acutely admitted patients.

Amyloid-beta (Aβ) peptides and hyperphosphorylated tau (p-tau) are key components of the extracellular amyloid plaques^19^ and NFTs respectively. The longer Aβ–peptides with 42 or more amino acids are prone to aggregate into amyloid plaques when the core is formed. Thus, low CSF concentration of the Aβ_1-42_ (Aβ42) peptide is commonly used to denote A pathology. The CSF concentrations of the shorter and less aggregation prone Aβ-peptides e.g., Aβ_1-40_ (Aβ40) is less affected by brain amyloid plaque formation. Adjusting for the overall Aβ –peptide release and sample handling by applying the Aβ42/40 ratio is suggested as a more accurate CSF biomarker of brain amyloid (A) pathology than Aβ42 itself.^20^

The aim of this study was to further explore the association between delirium in hip fracture patients with and without pre-fracture clinical dementia, and the AT(N) neuropathology by use of CSF biomarkers in a larger cohort than previously studied. The Aβ42/40 ratio was used to indicate brain A pathology, p-tau_181_ to indicate T pathology and total-tau to indicate N.^20^ Particularly the A and T pathologies in patients with no pre-fracture clinical dementia were of interest. We hypothesised that a CSF biomarker profile associated with AD would correlate with a higher risk of delirium.

## Methods

### Study participants

**Fig. 1:**
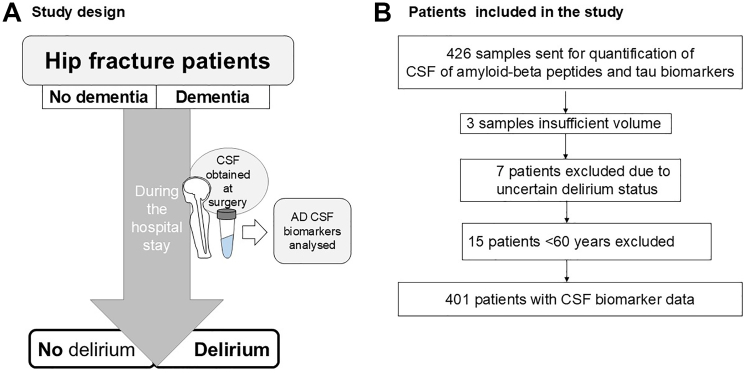
**Study design and patient inclusion**. **A)** The CSF concentrations of the AD biomarkers amyloid-beta_1-42_, phosphorylated tau_181_ and total-tau were analysed in hip fracture patients with or without delirium anytime during the hospital stay. The main analyses were performed separately in patients with and without dementia. CSF was obtained in connection with surgery. **B)** The final analysis was performed in 401 hip fracture patients with the required clinical data above 60 years.

We recruited patients with hip fracture from five Norwegian hospitals in the Oslo region from 2016 to 2020 as previously described.^21^ The patients were categorised by delirium (anytime during the hospital stay) and pre-fracture clinical dementia status (see Fig. 1). Pre-fracture dementia status was assessed using The Informant Questionnaire on Cognitive Decline in the Elderly (IQCODE) with a cut-off for dementia of ≥3.44.^22^ In case of missing IQCODE (n = 33) the pre-fracture dementia status was assessed using information from hospital records. Only patients >60 years were included in the AT(N) biomarker analyses since AD is uncommon at younger ages. Sex data were self-reported; the options provided were male or female.

We assessed delirium daily, preoperatively and until the fifth postoperative day, according to the Diagnostic and Statistical Manual of Mental Disorders–5 criteria,^1^ based on a standardised procedure previously described.^21^ The delirium diagnosis was based on interviews with the patients, supplemented by information from relatives, nurses and clinical notes. The patients were assessed with bedside tests of cognition that included the 4AT,^23^ OSLA,^24^ RASS and attention tests,^25^ The patients were also categorised according to dementia status (see Fig. 1). In summary the study included:

Patients without pre-fracture dementia:

I. No delirium (n = 181)
II. Delirium (n = 56)

Patients with pre-fracture dementia:

I. No delirium (n = 33)
II. Delirium (n = 131)

### CSF sampling and storage

Up to five millilitres of CSF were sampled from each patient before injection of spinal anaesthesia. In all patients, CSF was collected in sterile polypropylene tubes and centrifuged in room temperature for 10 min at 2000 G. The samples were aliquoted into 0.5 ml polypropylene tubes and stored at −80 °C. All CSF samples were sent on dry ice for biochemical analyses.

### Quantification of the CSF Aβ42, Aβ40, p–tau_181_ and t-tau

The concentrations of Aβ1-40, Aβ1-42, total-tau and p–tau_181_ were quantified by Lumipulse G assays (Fujirebo, Ghent, Belgium) at the Clinical Neurochemistry Laboratory at Sahlgrenska University Hospital, Mölndal, Sweden. The Lumipulse Gassays were previously evaluated with intraassay CVs in the range from 2.0 to 5.6, please see Gobom et al. for these and further details.^26^ Due to insufficient sample volume CSF Abeta_1-40_ could not be determined in one patient, CSF p-tau_181_ could not be determined in four patients and CSF t-tau could not be determined in 16 patients. Abeta_1-40_, Abeta_1-42_ and p-tau_181_ could be quantified in all measured samples. However, eight patients had t-tau concentrations below the detection limit (<150 pg/ml) and these were included with an imputed value of 75 pg/ml (50% of the detection limit). One extreme outlier had t-tau 14 SD above the mean and was removed. Sample analysis was performed by lab technicians blinded to dementia/delirium status and all other clinical information.

**Fig. 2:**
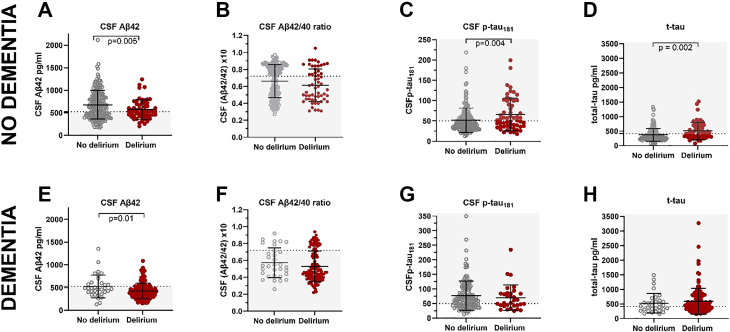
**CSF AD biomarkers in patients with no dementia (Fig. 2A–D, n** = **237) and in patients with pre-fracture dementia (Fig. 2E–H, n** = **164)**. The dotted lines represent cut-off values with the grey area indicating the biomarker positive values as according to laboratory-specific cut-offs. CSF Aβ 42/40 ratio presented as (CSF Aβ 1–42/CSF Aβ 1–40) x 10. Larger and smaller lines represent mean and standard deviation respectively, p-values are obtained by student's two sample t-test. No dementia: no delirium = 181, delirium = 56. Dementia: no delirium = 33, delirium = 131. CSF: cerebrospinal fluid, t-tau: total-tau.

The patients were dichotomised by CSF concentrations of the Aβ42/40 ratio ((Aβ42/40) x 10) and p–tau_181_ to categorise A and T status respectively. The laboratory specific cut-offs were A+ <0.72 and T+ >50 pg/ml and N+ >409 pg/ml. The Aβ42/40 ratio cut-off used to determine A status was set by use of patient samples analysed in routine laboratory setting utilising the clear bimodal distribution of this biomarker in patient samples while the p–tau_181_ and t-tau cut-offs was determined by use of three cohorts of non-demented controls and clinically diagnosed patients.^26^ All markers were also analysed as continuous markers, the cut-off for Aβ42 was <526 pg/ml, as indicated in Fig. 2.^26^

The concentrations of CSF p–tau_181_ and CSF t-tau correlated strongly (Pearsons correlation coeffecient 0.80 p < 0.001) and most patients (87%) had the same N status and T status. Of the AT(N) pathologies the primary aim was to study the AD specific AT neuropathologies. The N status was therefore not included in the combined categorical AT(N) analyses due to the overlap with the T pathology.

### Statistics

Continuous data were analysed by parametric tests as justified by the sample size according to the central limit theorem. The central tendency and spread of continuous biomarkers are therefore reported by mean and standard deviation and the group differences analysed by student's two sample t-test (student's t-test). The sample variation was assessed by Levene's test, and p-values reported according to the test outcome. No power calculations were performed as all available CSF samples were analysed.

Categorical variables were analysed by a Chi–Square (χ2) test in a 2 × 2 table and by logistic regressions to obtain odds-ratios and adjusted analyses. In the adjusted analyses age and gender were included based on statistical and biological grounds. Dementia was accounted for by stratified analyses of patients with and without dementia.

The significance level was set at α = 0.05 and all tests were two-tailed. In this exploratory study, we did not adjust for multiple hypothesis testing.

Statistical analyses were performed by use of the Statistical Package for Social Sciences (SPSS, v.28; IBM, Armonk, NY, USA). Graphical illustrations were created with GraphPad Prism (v.10.0.2 Graph Pad Software, La Jolla, CA, USA).

### Ethics

The study was performed in accordance with the Declaration of Helsinki. Informed consent was obtained from the patient or from the closest relative if the patient was unable to give consent. The study was approved by the Regional Committee for Ethics in Medical and Health Research in Norway (REK 2016/1368).

### Role of funders

The funders had no role in the design of the study, data collection, and analysis or preparation of the manuscript.

## Results

### The study population

In total 401 patients with hip fracture were included in the study of which half, (n = 187, 46.7%), had delirium during the hospital stay. Those with delirium were older than those with no delirium (85 years 95% confidence interval (CI) [84, 86] vs 78 years 95% CI [77, 79], p < 0.001). The proportion of female patients were similar among those with and without delirium (n = 127, 68% vs n = 143, 66%, χ^2^ p = 0.82). The characteristics of all patients with hip fracture with and without delirium in the full sample is outlined in Table 1 and the study setup in Fig. 1. The concentrations of the Aβ42 and Aβ40 peptides as well as the Aβ42/Aβ40 ratio (0.55, 95% CI [0.53, 0.58] vs 0.65 95% CI [0.62, 0.67], student's t-test, p < 0.001) were lower in patients with delirium. Brain Aβ-deposition (A+) was more common in patients with delirium (n = 138, 74% vs n = 115, 54%, χ^2^ p < 0.001). Patients with delirium had 35% higher concentrations of p-tau_181_ (73.9, 95% CI [67.0, 80.8] vs 54.6 95% CI [50.2, 59.1] pg/ml, student's t-test, p < 0.001) and 43% higher t-tau (563, 95% CI [504, 623] vs 394, 95% CI [369, 427] pg/ml, student's t-test, p < 0.001). Brain NFTs (T+) and neurodegeneration (N+) were more prevalent in delirium compared to those without delirium (n = 117, 63% vs n = 88, 43%, and n = 108, 60% vs n = 69, 34%, both p values < 0.001). In the full sample, all associations remained significant when adjusted for age and sex except for T+ (logistic regression, p = 0.06, see Table 2).

**Table 1:** Characteristics of all hip fracture patients.

All hip fracture patients with and without delirium										
	All			No delirium			Delirium			p-value
	*n*	Mean or n	Std.dev or %	*n*	Mean or n	Std.dev or %	*n*	Mean or n	Std.dev or %	No delirium vs delirium
Age, years^a^	*401*	81.4	(9.0)	*214*	78.3	(9.1)	*187*	85.0	(7.4)	<**0.001**
Sex (n, %)	*401*									
Female		270	(66.8)		143	(66.4)		127	(67.9)	0.82
Male		131	(33.2)		71	(33.5)		60	(32.1)	
IQCODE	*368*	3.62	(0.70)	*197*	3.26	(0.43)	*171*	4.04	(0.71)	<**0.001**
CSF biomarkers										
Amyloid-peptides										
Abeta_1-42_, pg/ml	*401*	569	(280)	*214*	655	(312)	*187*	471	(198)	<**0.001**
Abeta_1-40_, pg/ml	*400*	9579	(3539)	*213*	10167	(3554)	*187*	8909	(3410)	<**0.001**
Abeta_(1-42/1-40)_ x10	*400*	0.60	(0.20)	*213*	0.65	(0.19)	*187*	0.55	(0.19)	<**0.001**
A status^b^ (n, %)	*400*			*213*			*187*			
A−		147	(36.8)		98	(46.0)		49	(26.2)	<**0.001**
A+		253	(63.2)		115	(54.0)		138	(73.8)	
Tau-markers										
CSF p-tau_181_, pg/ml	*397*	63.7	(41.6)	*211*	54.63	(33.0)	*186*	73.9	(47.6)	<**0.001**
T status^c^ (n, %)	*397*									
T−		192	(48.4)		123	(58.3)		69	(37.1)	<**0.001**
T+		205	(51.6)		88	(42.9)		117	(62.9)	
CSF t-tau, pg/ml	*384*	473	(339)	*204*	394	(244)	*180*	563	(404)	<**0.001**
N-status^d^ (n, %)	384									
N−		207	(53.9)	*204*	135	(66.8)	*180*	72	(40.0)	<**0.001**
N+		177	(47.2)		69	(33.8)		108	(60.0)	

Categorical data are presented as number of cases (percentage) while continuous data are presented by mean (standard deviation). The reported p-values are obtained by Chi-square test (2 × 2 table) or student's two sample t-test for categorical and continuous variables respectively. Due to insufficient sample volume CSF Abeta_1-40_ could not be determined in one patient, CSF p-tau_181_ could not be determined in four patients and CSF t-tau could not be determined in 16 patients.

CSF: cerebrospinal fluid, p-tau_181_; Phosphorylated tau_181_; T-tau: total-tau; IQCODE Informant Questionnaire on Cognitive Decline in the Elderly.

p-values below 0.05 in bold.

a The highest and lowest age was 99 and 60 years both for patients with and without delirium.

b Amyloid positive (A+) by CSF Amyloid beta_1-42_/Amyloid beta_1-40_ ratio <0.72.

c T+ by CSF P-tau_181_ >50 pg/ml.

d N+ by t-tau >409 pg/ml.

**Table 2:** Delirium and Alzheimer's disease biomarkers, adjusted and unadjusted logistic regression.

	*N*	Unadjusted			Adjusted for age and sex		
		OR	95% CI for OR	p	OR	95% CI for OR	p
Full sample							
Abeta_1-42_	*401*	0.997	0.996–0.998	<**0.001**	0.997	0.996–0.998	<**0.001**
p-tau_181_	*397*	1.013	1.007–1.019	<**0.001**	1.007	1.001–1.013	**0.02**
t-tau	*377*	1.001	1.001–1.002	<**0.001**	1.001	1.000–1.001	**0.03**
(Aβ42/40) ×10	*400*	0.080	0.028–0.229	<**0.001**	0.207	0.066–0.644	**0.007**
A-status^a^	*400*	2.400	1.572–3.663	<**0.001**	1.742	1.103–2.750	**0.02**
T-status^b^	*397*	2.370	1.582–3.551	<**0.001**	1.522	0.977–2.373	0.06
N-status^c^	384	2935	1936–4449	<**0.001**	1915	1217–3014	**0.005**
A+T+^d^	*298*	3.120	1.919–5.073	<**0.001**	1.985	1.164–3.385	**0.01**
A+T+^e^	*397*	2.730	1.816–4.105	<**0.001**	1.875	1.207–2.914	**0.005**
No prefracture dementia							
Abeta_1-42_	*237*	0.999	0.997–0.9998	**0.02**	0.999	0.998–0.9999	**0.03**
p-tau_181_	*235*	1.012	1.003–1.020	**0.01**	1.006	0.997–1.015	0.18
t-tau	*227*	1.002	1.001–1.003	**0.001**	1.001	1.000–1.003	**0.046**
(Aβ42/40) ×10	*237*	0.259	0.055–1.223	0.09	0.632	0.122–3.276	0.59
A-status^a^	*237*	1.969	1.054–3.679	**0.03**	1.521	0.788–2.935	0.21
T-status^b^	235	1.797	0.981–3.290	0.06	1.117	0.572–2.178	0.75
N-status^c^	227	2.393	1.286–4452	**0.006**	1.476	0.744–2.927	0.27
A+T+^d^	169	2.375	1.149–4.909	**0.02**	1.508	0.674–3.370	0.32
A+T+^e^	235	2.006	1.085–3.709	**0.03**	1.370	0.705–2.660	0.35
Pre-fracture dementia							
Abeta_1-42_	164	0.998	0.996–0.999	**0.01**	0.998	0.996–1.000	**0.02**
p-tau_181_	162	1.003	0.994–1.01	0.46	1.000	0.991–1.009	0.93
t-tau	157	1.000	0.999–1.00	0.44	1.000	0.999–1.001	0.60
(Aβ42/40) ×10	163	0.278	0.034–2.250	0.23	0.600	0.064–5.613	0.65
A-status^a^	163	0.943	0.371–2.394	0.90	0.704	0.261–1.897	0.49
T-status^b^	162	1.574	0.715–3.461	0.26	1.223	0.529–2.828	0.64
N-status^c^	157	1.569	0.716–3.437	0.26	1.277	0.557–2.930	0.56
A+T+^d^	129	1.206	0.428–3.398	0.72	0.873	0.290–2.626	0.81
A+T+^e^	162	1.507	0.691–3.289	0.30	1.193	0.521–2.730	0.68

The table presents analyses of logistic regression in all hip fracture patients with dependent variable delirium during the hospital stay (delirium coded as 1, no delirium as 0, A+T+ coded as 1 while A−T− as 0). Due to insufficient sample volume CSF Abeta_1-40_ could not be determined in one patient, CSF p-tau_181_ could not be determined in four patients and CSF t-tau could not be determined in 16 patients.

OR: Odds-ratio, CI: Confidence Interval.

p-values below 0.05 in bold.

a Amyloid positive (A+) by CSF Amyloid beta_1-42_/Amyloid beta_1-40_ ratio <0.72.

b T+ by CSF p-tau_181_ >50 pg/ml.

c N+ by t-tau >409 pg/ml.

d A+T+ compared to those A−T−.

e A+T+ vs all others.

The patients with hip fracture with dementia (n = 164, 41%) were on average five years older than those without dementia (84, 95% CI [83, 86] years vs 79, 95% CI [78, 81] years). Those with dementia had, as expected, a lower Aβ42/Aβ40 ratio and had higher concentrations of p-tau_181_ and t-tau (student's t-test, all p-values < 0.001, values are given in Suppl Table S1). The analyses were therefore repeated stratified on dementia status.

### Amyloid-beta peptides (A status) and delirium

In patients without dementia those with delirium had lower CSF Aβ42 concentrations than those with no delirium (573 pg/ml 95% CI [515, 631], vs 679 pg/ml, 95% CI [632, 725] student's t-test p = 0.005, see Suppl Table S1 and Fig. 2). About half of these patients without dementia were A+, and such patients were more likely to develop delirium (n = 37, 66%, vs n = 90, 50%, χ^2^ p = 0.03, see Suppl Table S1). While the mean Aβ42/Aβ40 ratio was below the cut-off for A+ (<0.72) in both those with and without delirium, the mean CSF Aβ42 concentrations were in both groups above the cut-off (Aβ42 < 526 pg/ml).

Most patients with dementia were A+, regardless of delirium status (n = 126, 77%). The proportion of patients being A+ was similar in those with (77%) and without delirium (78%). Still, patients with delirium superimposed on dementia had lower CSF Aβ42 concentrations than those with no delirium (427 pg/ml 95% CI [397, 456] vs. 525 95% CI [437, 614] pg/ml, student's t-test p = 0.01, see Fig. 2 and Suppl Table S1).

### Tau markers (T and N status) and delirium

In patients without dementia, those with delirium had higher CSF p-tau_181_ concentrations (66 pg/ml 95% CI [56, 77] vs 52 pg/ml 95% CI [47, 56], student's t-test, p = 0.004 see Fig. 2 and Suppl Table S1). More than half of the patients without dementia were T-, indicating that they did not have AD related NFTs and tangle (T) pathology.^27^ The proportion of T+ was higher in those with delirium but, the significance level was not reached (n = 30, 54% vs n = 70, 39%, χ^2^ p = 0.06, Suppl Table S1). Patients without dementia but delirium also had higher CSF t-tau concentrations, indicating axonal neurodegeneration or acute damage,^27^ compared to those without delirium (508 pg/ml 95% CI [427, 589] vs 370 95% CI [337, 402] pg/ml, student's t-test, p = 0.002, see Fig. 2 and Suppl Table S1). The proportion of N+ was higher in those with delirium (n = 28, 51% vs n = 52, 30%, χ^2^ p = 0.006, see Suppl Table S1).

In those with dementia, delirium was associated with higher concentrations of both tau markers, and more were T+ (n = 87, 67% vs n = 18, 56%) and N+ (n = 80, 64% vs n = 17, 53%). However, none of these differences were statistically significant (see Fig. 2 and Suppl Table S1).

### AT status is associated with delirium in patients without pre-fracture dementia

**Fig. 3:**
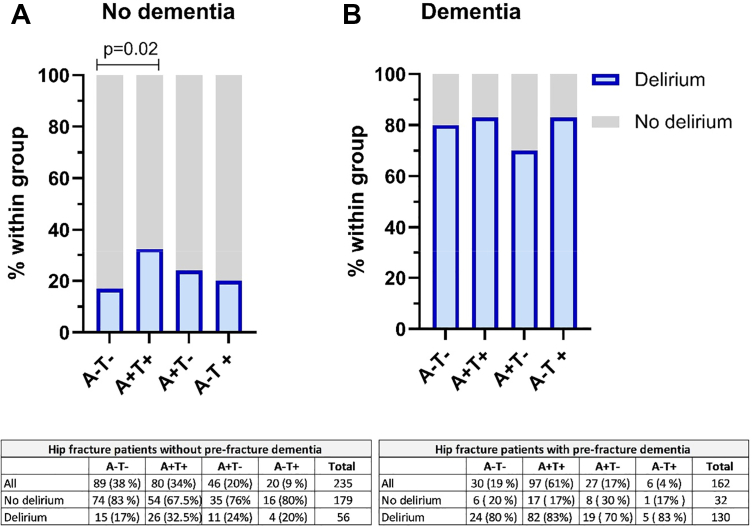
**AT biomarkers in patients with no pre-fracture dementia**. **A**) In patients without pre-fracture dementia delirium was more frequent among A+T+ patients than in those A−T− (p = 0.02) with number of patients (percentages) in each group below **B**) There were no statistically significant differences in the frequence of delirium across the four AT groups in hip fracture patients with pre-fracture dementia. The number of patients (percentages) in each group is presented below. Group differences were analysed by a 2 × 2 table Chi–Square test.

The patients were categorised according to the AT(N) classification^13^ into four groups A−T−, A+T−, A+T+ and A−T+, see Fig. 3. Most patients without dementia were either biomarker negative for both pathologies (A−T−, n = 89, 38%), or biomarker positive for both pathologies (A+T+, n = 80, 34%). The proportion of patients with delirium was lowest in the A−T− category (n = 15, 17%) and gradually increased to n = 4, 20% in the A−T+ group, n = 11, 24% in the A+/T− group, and reached the highest level in the A+/T+ group at n = 26, 34% (Fig. 3A). In patients with dementia, there were no differences in the frequency of delirium between those A+T+ and those A−T− (see Fig. 3B).

When the analyses stratified by dementia status were adjusted for age and sex, associations remained significant between delirium and lower Aβ42 concentrations (in both strata) and delirium and higher t-tau (only in those without dementia, Table 1). However, the associations between delirium and the other biomarkers were no longer significant.

## Discussion

In this cohort of more than 400 patients with hip fracture we found the AD AT(N) neuropathology, as indicated by CSF biomarkers, to associate with delirium. The associations with delirium were more pronounced in patients without clinical dementia. Collectively our study points to an association between AD AT neuropathology and delirium, and that such pathology increased the risk of delirium also in patients without clinical dementia.

Categorisation according to AT status showed that delirium was more common in those A+T+ compared to those A−T−. In large, we reiterate our previous findings in a smaller hip fracture cohort in which 65 of 129 patients did not have pre-fracture dementia.^15^ Fong and colleagues studied delirium in elective surgery patients without cognitive impairment, and found those positive for any of the AT(N) pathologies i.e., either A+ and/or T+ and/or N+ to have a higher, but not statistically significant, rate of delirium (p = 0.10).^18^ Albeit not significant for delirium occurrence which may be due to the limited size with few AT(N) positive delirium patients, their findings align with our findings of delirium being more prevalent in A+T+ hip fracture patients. Thus, our and previous studies support the hypothesis that brain AD AT pathology increases the vulnerability towards delirium, already in the preclinical phase.

Tau-pathologies relate more to cognitive decline and neurodegeneration than the Aβ-pathology.^28^, ^29^, ^30^ Being an acute cognitive disturbance it is not unexpected that delirium associates with the tau–markers. A more recent study including patients with an MMSE >23 in China analysed Aβ42 and the tau-markers as continuous markers and found an association with delirium.^31^ However, when our data was analysed stratified on pre-fracture dementia status, only lower Aβ42 concentrations remained significantly associated with delirium in both strata after adjustment for age and sex, suggesting that Aβ–peptides and deposition of these to be a stronger risk marker for delirium than AD related tau–pathology. This was also concluded by Cunningham and colleagues in their study of elective surgery patients without dementia.^16^ The study by Cunningham and colleagues was however performed prior to common use of AT(N) status and co-existing T pathology among those with A+ may have confounded the results. In the study by Fong and colleagues, the majority of their AT(N) positive patients were A+, supporting that brain amyloid-beta deposition is associated with an increased risk of delirium. Thus, our data gives further support to the hypothesis of an association between A pathology and delirium. We speculate this to be caused by neuroinflammation induced by the disease associated microglia (DAM) phenotype in plaque surrounded microglia and vascular components of amyloid-beta deposition. The incomplete understanding of the CSF dynamics of the tau markers should however be kept in mind.^27^

In patients with dementia the only significant association with delirium we observed was lower CSF Aβ42 concentrations. Dementia is among the strongest risk factors for delirium^2^^,^^3^ and as expected most patients with dementia developed delirium (80%). Also as expected, the majority (>80%) of patients with dementia had biomarker evidence of A and/or T pathology. Those without biomarker evidence of A and/or T pathology could have other pathologies e.g. vascular also increasing the risk for delirium. Since both delirium and CSF biomarker pathology are extremely common in patients with dementia, it is harder to study associations between the two in such patients. Therefore, the results in this patient population should be interpreted with caution.

Strengths of this study includes a fairly large number of patients with and without dementia. To our knowledge this is the first study to use the Aβ42/Aβ40 ratio to determine A-status in delirium, which is a superior CSF measure to indicate brain Aβ–deposition than Aβ42 alone. Our study focuses on acutely admitted hip fracture patients, a population characterised by high rates of frailty, multimorbidity, and underlying brain pathology, even in the absence of clinical dementia. While including patients and sampling CSF is more challenging in acute settings compared to elective surgeries, the higher rates of delirium in these patients represent a significant strength of our study.

Study limitations include that pre-fracture dementia status was based on the IQCODE which, whilst validated and commonly used,^32^ is not a substitute for objective cognitive testing or a complete dementia assessment. Another limitation is the absence of adjustments for other risk factors that are likely to influence the outcomes, such as hypertension, diabetes, vascular risk factors, education, body mass index (BMI), polypharmacy and APOE4 status. We acknowledge that the lack of adjustment for multiple hypothesis testing is a limitation of our exploratory study design. This may increase the risk of Type I error and should be considered when interpreting our findings.

The AD AT(N) CSF biomarkers associated with delirium, especially in patients without clinical dementia. This indicates the AD AT(N) pathology to be a risk factor for delirium, also in stages preceding clinical dementia. We found that amyloid pathology, in particular, was associated with an increased risk of delirium. These findings support the rationale for conducting a drug trial to evaluate whether anti-amyloid therapy could reduce delirium risk. For future work, it would also be valuable to investigate whether different AT(N) combinations are associated with varying degrees of delirium risk based on distinct precipitating factors.

## Contributors

KH and LOW conceptualised the study. KH, BEN, KB, CTP, RBO, LS, MM, OTØ, AK, AG, AVI, and LOW curated the data. KB and HZ provided the biochemical analyses. LOW and KH acquired funding. LOW administered and supervised the project. KH and LOW wrote the original draft. LOW and KH had access and verified the underlying data. All authors reviewed and edited the manuscript and read and approved the final version of the manuscript.

## Data sharing statement

Owing to ethical restrictions, the full dataset is available to the reader upon request only. Proposals should be directed to the corresponding author at l.o.watne@medisin.uio.no and to gain access, data requestors will need to sign a data access agreement.

## Declaration of interests

BEN is funded by South-Eastern Norway Regional Health Authorities (#2018070 and 2024017).

KB was during the performance of this research supported by the Swedish Research Council (#2022-00732), the Swedish Alzheimer Foundation (#AF-994551), Hjärnfonden, Sweden (#FO2024-0048-TK-130 and FO2024-0048-HK-24), and the Swedish state under the agreement between the Swedish government and the County Councils, the ALF-agreement (#ALFGBG-1006418).

KB has during 2024–2025 served as a consultant, at advisory boards, and has given lectures, produced educational materials and participated in educational programs for AC Immune, ALZPath, AriBio, Beckman–Coulter, BioArctic, Eisai, Lilly, Neurimmune, Novartis, Roche Diagnostics, Sunbird Bio, and Siemens Healthineers; all before Sept 2026. KB is a co-founder of Brain Biomarker Solutions in Gothenburg AB (BBS), which is a part of the GU Ventures Incubator Program, outside the work presented in this paper. KB has since Sept 2025 been an employee of Lilly.

HZ is a Wallenberg Scholar and a Distinguished Professor at the Swedish Research Council supported by grants from the Swedish Research Council (#2023-00356, #2022-01018 and #2019-02397), the European Union's Horizon Europe research and innovation programme under grant agreement No 101053962, Swedish State Support for Clinical Research (#ALFGBG-71320), the Alzheimer Drug Discovery Foundation (ADDF), USA (#201809-2016862), the AD Strategic Fund and the Alzheimer's Association (#ADSF-21-831376-C, #ADSF-21-831381-C, #ADSF-21-831377-C, and #ADSF-24-1284328-C), the European Partnership on Metrology, co-financed from the European Union's Horizon Europe Research and Innovation Programme and by the Participating States (NEuroBioStand, #22HLT07), the Bluefield Project, Cure Alzheimer's Fund, the Olav Thon Foundation, the Erling-Persson Family Foundation, Familjen Rönströms Stiftelse, Stiftelsen för Gamla Tjänarinnor, Hjärnfonden, Sweden (#FO2022-0270), the European Union's Horizon 2020 research and innovation programme under the Marie Skłodowska-Curie grant agreement No 860197 (MIRIADE), the European Union Joint Programme – Neurodegenerative Disease Research (JPND2021-00694), the National Institute for Health and Care Research University College London Hospitals Biomedical Research Centre, and the UK Dementia Research Institute at UCL (UKDRI-1003).

HZ has served at scientific advisory boards and/or as a consultant for Abbvie, Acumen, Alector, Alzinova, ALZpath, Amylyx, Annexon, Apellis, Artery Therapeutics, AZTherapies, Cognito Therapeutics, CogRx, Denali, Eisai, LabCorp, Merry Life, Nervgen, Novo Nordisk, Optoceutics, Passage Bio, Pinteon Therapeutics, Prothena, Quanterix, Red Abbey Labs, reMYND, Roche, Samumed, Siemens Healthineers, Triplet Therapeutics, and Wave, has given lectures sponsored by Alzecure, BioArctic, Biogen, Cellectricon, Fujirebio, Lilly, Novo Nordisk, Roche, and WebMD, and is a co-founder of Brain Biomarker Solutions in Gothenburg AB (BBS), which is a part of the GU Ventures Incubator Program (outside submitted work).
