# Supplementary material for: Biomarkers of Alzheimer's disease pathophysiology and delirium
Source: eBioMedicine. 2026 Apr 10;127:106252. doi: 10.1016/j.ebiom.2026.106252 (PMC13091946; doi:10.1016/j.ebiom.2026.106252)
Supplement: Supplementary Table [file mmc1.docx]

**Supplementary Table 1 Characteristics of the hip fracture patients stratified by dementia status**

| **Patients without pre-fracture dementia** | | | | | | | | | | | | |
| --- | --- | --- | --- | --- | --- | --- | --- | --- | --- | --- | --- | --- |
|  |  | **All** |  | **No delirium** | | | | **Delirium** | | | | **p-value** |
|  | *n* | mean or n | std.dev or % | *n* | mean or n | std.dev or % | | *n* | | Mean or n | std.dev or % |  |
| Age, years | *237* | 79.3 | (9.3) | *181* | 77.9 | (9.0) | | *56* | | 83.9 | (9.0) | **p<0.001** |
| Sex (n, %) | *237* |  |  | *181* |  |  | | *56* | |  |  |  |
| Female |  | 158 | (66.7) |  | 122 | (67.4) | |  | | 36 | (64.3) | 0.67 |
| Male |  | 79 | (33.3) |  | 59 | (32.6) | |  | | 20 | (35.7) |  |
| IQCODE | *214* | 3.11 | (0.13) | *164* | 3.09 | (0.12) | | *50* | | 3.19 | (0.14) | **p<0.001** |
|  |  |  |  |  |  |  | |  | |  |  |  |
| **CSF biomarkers** |  |  |  |  |  |  | |  | |  |  |  |
| *Amyloid-peptides* |  |  |  |  |  |  | |  | |  |  |  |
| CSF Abeta_1-42_ ,pg/ml | *237* | 654 | (300) | *181* | 679 | (317) | | *56* | | 573 | 216 | **0.005^d^** |
| CSF Abeta_1-40,_ pg/ml | *237* | 10190 | (3618) | *181* | 10257 | (3540) | | *56* | | 9973 | (3885) | 0.61 |
| CSF Abeta (_1-42/1-40_) x10 | *237* | 0.65 | (0.19) | *181* | 0.66 | (0.19) | | *56* | | 0.61 | (0.20) | 0.09 |
| A status^a^ (n, %) | *237* |  |  | *181* |  |  | | *56* | |  |  |  |
| A- |  | 110 | (46.4) |  | 91 | (50.3) | |  | | 19 | (33.9) | **0.03** |
| A+ |  | 127 | (53.6) |  | 90 | (49.7) | |  | | 37 | (66.1) |  |
| *Tau markers* |  |  |  |  |  |  | |  | |  |  |  |
| CSF p-tau_181_, pg/ml | *235* | 55.3 | (33.1) | *179* | 51.9 | (29.9) | | *56* | | 66.2 | (40.0) | **0.004** |
| T status^b^ (n, %) | *235* |  |  | *179* |  |  | | *56* | |  |  |  |
| T- |  | 135 | (57.4) |  | 109 | (60.9) | |  | | 26 | (46.4) | 0.056 |
| T+ |  | 100 | (42.6) |  | 70 | (39.1) | |  | | 30 | (53.6) |  |
| CSF t-tau, pg/ml | *227* | 403 | (316) | *172* | 370 | (216) | | *55* | | 508 | (299) | **0.002** |
| N status^c^ (n, %) | *227* |  |  |  |  |  | |  | |  |  |  |
| N- |  | 147 | (64.8) |  | 120 | (69.8) | |  | | 27 | (49.1) | **0.006** |
| N+ |  | 80 | (35.2) |  | 52 | (30.2) | |  | | 28 | (50.9) |  |
| **Patients with pre-fracture dementia** | | | | | | | | | | | | |
|  |  | **All** |  | **No delirium** | | | | **Delirium** | | | | **p-value** |
|  | *n* |  |  | *n* |  |  | | | *n* |  |  |  |
| Age, years | *164* | 84.4 | (7.5) | *33* | 80.1 | (9.3) | | | *131* | 85.5 | (6.6) | **p<0.001** |
| Sex (n, %) |  |  |  |  |  |  | | |  |  |  |  |
| Female |  | 112 | (68.3) |  | 21 | (62.5) | | |  | 91 | (69.2) | 0.67 |
| Male |  | 52 | (31.7) |  | 12 | (37.5) | | |  | 40 | (30.8) |  |
| IQCODE | *154* | 4.33 | (0.53) | *33* | 4.09 | (0.47) | | | *121* | 4.39 | (0.52) | **0.003** |
|  |  |  |  |  |  |  | | |  |  |  |  |
| **CSF biomarkers** |  |  |  |  |  |  | | |  |  |  |  |
| *Amyloid-peptides* |  |  |  |  |  |  | | |  |  |  |  |
| CSF Abeta_1-42_, pg/ml | *164* | 447 | (193) | *33* | 525 | (249) | | | *131* | 427 | (172) | **0.01** |
| CSF Abeta_1-40,_ pg/ml | *163* | 8690 | (3231) | *32* | 9657 | (3646) | | | *131* | 8454 | (3091) | 0.06 |
| CSF Abeta (_1-42/1-40_) x10 | *163* | 0.54 | (0.18) | *32* | 0.57 | (0.18) | | | *131* | 0.53 | (0.18) | 0.23 |
| A status ^a^ | *163* |  |  | *32* |  |  | | | *131* |  |  |  |
| A- |  | 37 | (22.7) |  | 7 | (21.9) | | |  | 30 | (22.9) | 0.90 |
| A+ |  | 126 | (77.3) |  | 25 | (78.1) | | |  | 101 | (77.1) |  |
| *Tau markers* |  |  |  |  |  |  | | |  |  |  |  |
| CSF p-tau_181_ pg/ml | *162* | 75.8 | (49.1) | *32* | 70.1 | (44.0) | | | *130* | 77.3 | (50.3) | 0.46 |
| T status ^b^ | *162* |  |  |  |  |  | | |  |  |  |  |
| T- |  | 57 | (35.2) |  | 14 | (43.8) | | |  | 43 | (33.1) | 0.26 |
| T+ |  | 105 | (64.8) |  | 18 | (56.3) | | |  | 87 | (66.9) |  |
| CSF t-tau pg/ml | *157* | 578 | (421) | *32* | 523 | (334) | | | *125* | 588 | (442) | 0.44 |
| N status ^c^ | *157* |  |  |  |  |  |  | | |  |  |  |
| N- |  | 60 | (38.2) |  | 15 | (46.9) |  | | | 45 | (36.0) | 0.31 |
| N+ |  | 97 | (61.8) |  | 17 | (53.1) |  | | | 80 | (64.0) |  |

Categorical data are presented as number of cases (percentage) with p-values (no delirium vs delirium) obtained by Chi-square test (2x2 table). Continuous data are presented as mean (standard deviation) with p –values (no delirium vs delirium) obtained by student’s two sample t-test. Due to insufficient sample volume CSF Abeta_1-40_ could not be determined in one patient, CSF p-tau_181_ could not be determined in four patients and CSF t-tau could not be determined in 16 patients. *CSF: cerebrospinal fluid, p-tau_181_; Phosphorylated tau_181_; T-tau: total-tau; IQCODE* *Informant Questionnaire on Cognitive Decline in the Elderly.*

a Amyloid positive (A+) by CSF Amyloid beta _1-42_/Amyloid beta _1-40_ ratio < 0.72

b T+ by CSF p-tau_181_ > 50 pg/ml

c N+ by t-tau > 409 pg/ml

d Levenes's test not passed, p-value reported not assuming equal variances.
